# *N*,*N*-Dibenzyl-4-methyl­benzene­sulfonamide

**DOI:** 10.1107/S1600536810015059

**Published:** 2010-04-30

**Authors:** Islam Ullah Khan, Waqar Ahmad, Shahzad Sharif, Salamat Ali, Edward R. T. Tiekink

**Affiliations:** a Materials Chemistry Laboratory, Department of Chemistry, Government College, University, Lahore 54000, Pakistan; b Department of Physics, Government College University, Lahore 54000, Pakistan; c Department of Chemistry, University of Malaya, 50603 Kuala Lumpur, Malaysia

## Abstract

The asymmetric unit of the title compound, C_21_H_21_NO_2_S, comprises two mol­ecules with similar conformations. The benzene rings of the nitro­gen-bound benzyl groups lie to the same side of the mol­ecule but are splayed in opposite directions precluding π–π inter­actions between them. In the crystal, each independent mol­ecule self-associates *via* inter­molecular C—H⋯O inter­actions, forming a supra­molecular chain propagating along the *b* axis.

## Related literature

For related structures, see: Khan *et al.* (2010 ▶); Arshad *et al.* (2009 ▶).
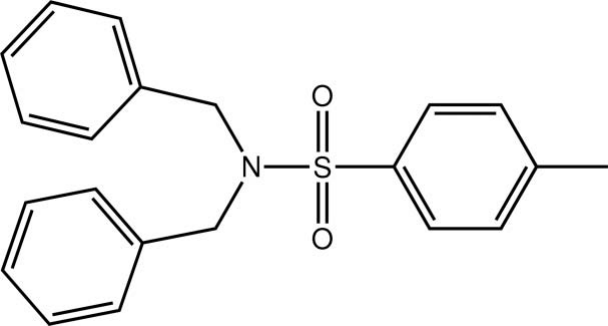


## Experimental

### 

#### Crystal data

C_21_H_21_NO_2_S
*M* *_r_* = 351.45
Orthorhombic, 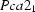

*a* = 27.7716 (15) Å
*b* = 5.9523 (3) Å
*c* = 22.3140 (12) Å
*V* = 3688.6 (3) Å^3^
*Z* = 8
Mo *K*α radiation
μ = 0.19 mm^−1^
*T* = 293 K
0.39 × 0.11 × 0.07 mm

#### Data collection

Bruker APEXII CCD diffractometer
51453 measured reflections
6474 independent reflections
3191 reflections with *I* > 2σ(*I*)
*R*_int_ = 0.122

#### Refinement

*R*[*F* ^2^ > 2σ(*F* ^2^)] = 0.062
*wR*(*F* ^2^) = 0.232
*S* = 1.02
6474 reflections
453 parameters
1 restraint
H-atom parameters constrained
Δρ_max_ = 0.31 e Å^−3^
Δρ_min_ = −0.35 e Å^−3^
Absolute structure: Flack (1983 ▶), 3144 Friedel pairs
Flack parameter: 0.18 (17)

### 

Data collection: *APEX2* (Bruker, 2007 ▶); cell refinement: *SAINT* (Bruker, 2007 ▶); data reduction: *SAINT*; program(s) used to solve structure: *SHELXS97* (Sheldrick, 2008 ▶); program(s) used to refine structure: *SHELXL97* (Sheldrick, 2008 ▶); molecular graphics: *ORTEP-3* (Farrugia, 1997 ▶), *QMol* (Gans & Shalloway, 2001 ▶) and *DIAMOND* (Brandenburg, 2006 ▶); software used to prepare material for publication: *publCIF* (Westrip, 2010 ▶).

## Supplementary Material

Crystal structure: contains datablocks global, I. DOI: 10.1107/S1600536810015059/hb5415sup1.cif

Structure factors: contains datablocks I. DOI: 10.1107/S1600536810015059/hb5415Isup2.hkl

Additional supplementary materials:  crystallographic information; 3D view; checkCIF report

## Figures and Tables

**Table 1:** Hydrogen-bond geometry (Å, °)

*D*—H⋯*A*	*D*—H	H⋯*A*	*D*⋯*A*	*D*—H⋯*A*
C8—H8a⋯O1^i^	0.97	2.58	3.456 (9)	151
C36—H36a⋯O4^i^	0.97	2.51	3.404 (9)	154

Symmetry code: (i) 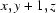.

**Fig. 1.**
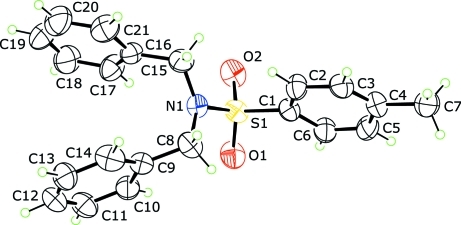
The molecular structure of the first independent molecule in (I) showing displacement ellipsoids at the 35% probability level.

**Fig. 2.**
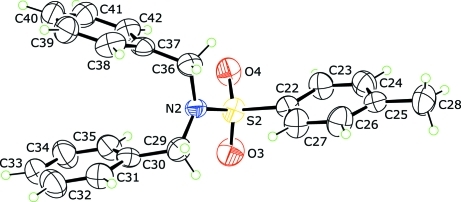
The molecular structure of the second independent molecule in (I) showing displacement ellipsoids at the 35% probability level.

**Fig. 3.**
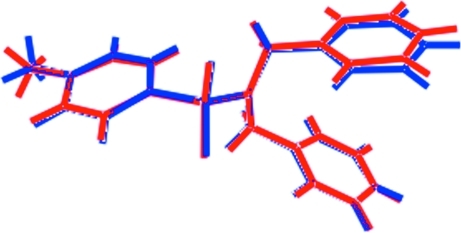
Overlap diagram highlighting the different conformations of the first (red) and second (blue) independent molecules in (I).

**Fig. 4.**
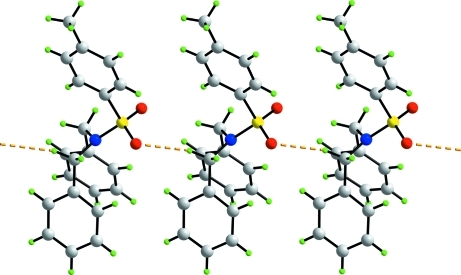
A view of a supramolecular chain in (I) formed by the first independent molecule aligned along the b axis. The C–H···O contacts are shown as orange dashed lines. Colour code: S, yellow; O, red; N, blue; C, grey; and H, green.

## supplementary crystallographic information

### Comment

The title compound, (I), was investigated as an extension of previously reported studies (Khan *et al.*, 2010; Arshad *et al.*, 2009). Two independent molecules comprise the crystallographic asymmetric unit of (I), Figs 1 and 2. The conformations are very similar to each other with the inverted form of the first molecule being virtually superimposable on the second independent molecule, Fig. 3. This similarity is quantified in the r.m.s. values for bond distances and angles of 0.0316 Å and 1.717 °, respectively. In terms of the molecular conformation, the benzene rings of the benzyl groups are orientated in the same direction but are splayed somewhat so that there is no evidence of a π–π interaction between them [the dihedral angle between the C9–C14 and C16–C21 rings is 33.6 (5) °; 28.5 (5) ° for the dihedral angle between C30–C35 and C37–C42 rings in the second independent molecule]. The tolyl group is twisted out of the putative mirror plane bisecting the benzyl rings and containing the *N*–*S*–C_tolyl_ moiety as seen in the values of the N1–S1–C1–C2 and N2–S2–C22–C23 torsion angles of -27.9 (7) and -147.7 (6) °, respectively.

Each independent molecule self-associates into a supramolecular chain along the *b* axis that is sustained by C–H···O contacts, Table 1. A view of one such chain is shown in Fig. 4.

### Experimental

A mixture of *N*-benzyl-4-methylbenzenesulfonamide (0.5 g, 2.02 mmol), sodium hydride (0.2 g, 8.333 mmol) and *N*,*N*-dimethylformamide (10 ml) was stirred at room temperature for 30 min. followed by the addition of benzyl chloride (0.23 ml, 2.02 mmol). After complete consumption of reactants (as monitored by TLC), the contents were poured over crushed ice. The precipitated product was isolated, washed and recrystallized from methanol solution to yield colourless blocks of (I).

### Refinement

The H atoms were geometrically placed (C–H = 0.93–0.97 Å) and refined as riding with *U_iso_*(H) = 1.2-1.5*U_eq_*(C).

### Crystal data

**Table:**

C_21_H_21_NO_2_S	*F*(000) = 1488
*M**_r_* = 351.45	*D*_x_ = 1.266 Mg m^−^^3^
Orthorhombic, *P**c**a*2_1_	Mo *K*α radiation, λ = 0.71073 Å
Hall symbol: P 2c -2ac	Cell parameters from 1938 reflections
*a* = 27.7716 (15) Å	θ = 2.3–23.9°
*b* = 5.9523 (3) Å	µ = 0.19 mm^−^^1^
*c* = 22.3140 (12) Å	*T* = 293 K
*V* = 3688.6 (3) Å^3^	Block, colourless
*Z* = 8	0.39 × 0.11 × 0.07 mm

### Data collection

**Table:**

Bruker APEXII CCD diffractometer	3191 reflections with *I* > 2σ(*I*)
Radiation source: fine-focus sealed tube	*R*_int_ = 0.122
graphite	θ_max_ = 25.0°, θ_min_ = 1.5°
φ and ω scans	*h* = −33→33
51453 measured reflections	*k* = −7→6
6474 independent reflections	*l* = −26→26

### Refinement

**Table:**

Refinement on *F*^2^	Secondary atom site location: difference Fourier map
Least-squares matrix: full	Hydrogen site location: inferred from neighbouring sites
*R*[*F*^2^ > 2σ(*F*^2^)] = 0.062	H-atom parameters constrained
*wR*(*F*^2^) = 0.232	*w* = 1/[σ^2^(*F*_o_^2^) + (0.1204*P*)^2^] where *P* = (*F*_o_^2^ + 2*F*_c_^2^)/3
*S* = 1.02	(Δ/σ)_max_ < 0.001
6474 reflections	Δρ_max_ = 0.31 e Å^−^^3^
453 parameters	Δρ_min_ = −0.35 e Å^−^^3^
1 restraint	Absolute structure: Flack (1983), 3144 Friedel pairs
Primary atom site location: structure-invariant direct methods	Flack parameter: 0.18 (17)

### Special details

**Table:**

Geometry. All s.u.'s (except the s.u. in the dihedral angle between two l.s. planes) are estimated using the full covariance matrix. The cell s.u.'s are taken into account individually in the estimation of s.u.'s in distances, angles and torsion angles; correlations between s.u.'s in cell parameters are only used when they are defined by crystal symmetry. An approximate (isotropic) treatment of cell s.u.'s is used for estimating s.u.'s involving l.s. planes.
Refinement. Refinement of *F*^2^ against ALL reflections. The weighted *R*-factor wR and goodness of fit *S* are based on *F*^2^, conventional *R*-factors *R* are based on *F*, with *F* set to zero for negative *F*^2^. The threshold expression of *F*^2^ > 2σ(*F*^2^) is used only for calculating *R*-factors(gt) etc. and is not relevant to the choice of reflections for refinement. *R*-factors based on *F*^2^ are statistically about twice as large as those based on *F*, and *R*- factors based on ALL data will be even larger.

### Fractional atomic coordinates and isotropic or equivalent isotropic displacement parameters (Å^2^)

**Table:**

	*x*	*y*	*z*	*U*_iso_*/*U*_eq_	
S1	0.92190 (7)	0.3770 (3)	0.10735 (10)	0.0630 (6)	
O1	0.9371 (2)	0.2775 (9)	0.0517 (3)	0.0870 (19)	
O2	0.9176 (2)	0.2351 (9)	0.1592 (3)	0.0848 (17)	
N1	0.95928 (19)	0.5761 (9)	0.1242 (3)	0.0587 (17)	
C1	0.8657 (2)	0.5026 (11)	0.0928 (3)	0.058 (2)	
C2	0.8521 (3)	0.6993 (12)	0.1184 (4)	0.070 (2)	
H2	0.8730	0.7759	0.1438	0.084*	
C3	0.8066 (3)	0.7853 (13)	0.1061 (4)	0.069 (2)	
H3	0.7982	0.9257	0.1211	0.083*	
C4	0.7749 (3)	0.6720 (14)	0.0735 (4)	0.071 (2)	
C5	0.7891 (3)	0.4704 (15)	0.0470 (4)	0.079 (2)	
H5	0.7678	0.3908	0.0229	0.095*	
C6	0.8337 (3)	0.3923 (14)	0.0565 (4)	0.076 (2)	
H6	0.8432	0.2596	0.0379	0.091*	
C7	0.7245 (3)	0.7664 (17)	0.0596 (4)	0.098 (3)	
H7A	0.7007	0.6815	0.0811	0.148*	
H7B	0.7184	0.7556	0.0174	0.148*	
H7C	0.7230	0.9210	0.0717	0.148*	
C8	0.9737 (3)	0.7298 (12)	0.0754 (4)	0.073 (2)	
H8A	0.9625	0.8801	0.0847	0.087*	
H8B	0.9582	0.6827	0.0385	0.087*	
C9	1.0275 (3)	0.7360 (11)	0.0659 (3)	0.059 (2)	
C10	1.0507 (3)	0.5595 (13)	0.0392 (4)	0.073 (2)	
H10	1.0333	0.4332	0.0275	0.088*	
C11	1.0993 (4)	0.5681 (16)	0.0296 (4)	0.086 (3)	
H11	1.1150	0.4459	0.0124	0.103*	
C12	1.1244 (4)	0.752 (2)	0.0447 (5)	0.089 (3)	
H12	1.1573	0.7560	0.0367	0.107*	
C13	1.1041 (4)	0.9255 (19)	0.0706 (5)	0.098 (3)	
H13	1.1225	1.0488	0.0819	0.117*	
C14	1.0545 (4)	0.9232 (12)	0.0808 (4)	0.084 (3)	
H14	1.0396	1.0478	0.0978	0.101*	
C15	0.9642 (3)	0.6567 (13)	0.1858 (4)	0.073 (2)	
H15A	0.9418	0.5757	0.2112	0.087*	
H15B	0.9557	0.8146	0.1872	0.087*	
C16	1.0142 (3)	0.6280 (13)	0.2104 (3)	0.062 (2)	
C17	1.0412 (3)	0.4396 (14)	0.2004 (4)	0.084 (3)	
H17	1.0289	0.3236	0.1771	0.101*	
C18	1.0867 (4)	0.4208 (19)	0.2247 (5)	0.105 (3)	
H18	1.1054	0.2951	0.2162	0.126*	
C19	1.1038 (4)	0.579 (2)	0.2598 (5)	0.107 (3)	
H19	1.1340	0.5601	0.2773	0.129*	
C20	1.0794 (5)	0.762 (2)	0.2706 (5)	0.112 (4)	
H20	1.0930	0.8742	0.2942	0.135*	
C21	1.0328 (4)	0.7916 (15)	0.2468 (4)	0.092 (3)	
H21	1.0151	0.9197	0.2557	0.110*	
S2	0.61516 (7)	−0.1198 (3)	0.85180 (11)	0.0668 (6)	
O3	0.6084 (2)	−0.2503 (10)	0.7995 (3)	0.099 (2)	
O4	0.6322 (2)	−0.2288 (9)	0.9045 (3)	0.0878 (18)	
N2	0.65232 (19)	0.0765 (9)	0.8363 (2)	0.0564 (16)	
C22	0.5594 (3)	0.0061 (12)	0.8705 (3)	0.061 (2)	
C23	0.5302 (3)	−0.0963 (13)	0.9120 (4)	0.080 (3)	
H23	0.5399	−0.2277	0.9310	0.096*	
C24	0.4862 (3)	−0.0013 (16)	0.9251 (4)	0.086 (3)	
H24	0.4664	−0.0718	0.9529	0.103*	
C25	0.4706 (3)	0.1944 (15)	0.8985 (4)	0.072 (2)	
C26	0.5017 (3)	0.2958 (14)	0.8591 (5)	0.090 (3)	
H26	0.4927	0.4304	0.8411	0.108*	
C27	0.5448 (3)	0.2057 (13)	0.8459 (4)	0.083 (3)	
H27	0.5652	0.2805	0.8194	0.100*	
C28	0.4230 (3)	0.297 (2)	0.9128 (5)	0.117 (4)	
H28A	0.4278	0.4323	0.9354	0.176*	
H28B	0.4042	0.1929	0.9360	0.176*	
H28C	0.4063	0.3313	0.8763	0.176*	
C29	0.6577 (3)	0.1596 (13)	0.7761 (4)	0.073 (2)	
H29A	0.6498	0.3184	0.7757	0.088*	
H29B	0.6347	0.0832	0.7506	0.088*	
C30	0.7073 (3)	0.1295 (13)	0.7501 (3)	0.060 (2)	
C31	0.7283 (4)	0.2922 (14)	0.7144 (4)	0.081 (3)	
H31	0.7115	0.4240	0.7064	0.097*	
C32	0.7737 (4)	0.2633 (19)	0.6904 (5)	0.105 (3)	
H32	0.7868	0.3728	0.6657	0.125*	
C33	0.7993 (4)	0.071 (2)	0.7034 (5)	0.102 (3)	
H33	0.8304	0.0529	0.6888	0.122*	
C34	0.7786 (4)	−0.0930 (19)	0.7379 (5)	0.103 (3)	
H34	0.7953	−0.2252	0.7458	0.124*	
C35	0.7336 (3)	−0.0629 (14)	0.7607 (4)	0.080 (3)	
H35	0.7202	−0.1757	0.7842	0.096*	
C36	0.6680 (3)	0.2290 (12)	0.8854 (4)	0.070 (2)	
H36A	0.6577	0.3809	0.8764	0.084*	
H36B	0.6526	0.1835	0.9225	0.084*	
C37	0.7209 (3)	0.2269 (13)	0.8937 (4)	0.063 (2)	
C38	0.7496 (4)	0.4127 (12)	0.8793 (4)	0.083 (3)	
H38	0.7350	0.5442	0.8658	0.099*	
C39	0.7983 (4)	0.4037 (18)	0.8846 (5)	0.096 (3)	
H39	0.8163	0.5284	0.8735	0.115*	
C40	0.8216 (4)	0.218 (2)	0.9058 (5)	0.098 (3)	
H40	0.8550	0.2138	0.9087	0.118*	
C41	0.7942 (4)	0.0384 (16)	0.9226 (4)	0.088 (3)	
H41	0.8090	−0.0887	0.9383	0.105*	
C42	0.7448 (3)	0.0437 (12)	0.9164 (4)	0.071 (2)	
H42	0.7270	−0.0814	0.9280	0.086*	

### Atomic displacement parameters (Å^2^)

**Table:**

	*U*^11^	*U*^22^	*U*^33^	*U*^12^	*U*^13^	*U*^23^
S1	0.0686 (13)	0.0448 (9)	0.0757 (14)	0.0117 (9)	0.0100 (11)	0.0011 (12)
O1	0.079 (4)	0.076 (4)	0.106 (5)	0.013 (3)	0.006 (4)	−0.039 (4)
O2	0.083 (4)	0.061 (3)	0.110 (5)	0.006 (3)	0.008 (3)	0.019 (4)
N1	0.056 (4)	0.049 (3)	0.071 (5)	−0.001 (3)	−0.001 (3)	0.005 (3)
C1	0.056 (5)	0.047 (4)	0.070 (6)	0.006 (4)	0.006 (4)	−0.004 (4)
C2	0.058 (5)	0.067 (5)	0.084 (6)	0.004 (4)	−0.008 (5)	−0.021 (5)
C3	0.070 (6)	0.062 (4)	0.076 (6)	0.028 (4)	0.007 (5)	−0.005 (5)
C4	0.055 (5)	0.072 (5)	0.086 (6)	0.003 (4)	0.004 (5)	0.000 (5)
C5	0.069 (6)	0.093 (6)	0.076 (6)	−0.007 (5)	−0.012 (4)	−0.027 (5)
C6	0.066 (6)	0.077 (5)	0.084 (6)	0.008 (5)	0.000 (5)	−0.031 (5)
C7	0.083 (7)	0.128 (7)	0.084 (7)	0.035 (6)	−0.004 (5)	0.011 (6)
C8	0.070 (6)	0.058 (5)	0.090 (6)	0.011 (4)	0.010 (5)	0.017 (5)
C9	0.070 (6)	0.046 (4)	0.062 (5)	0.002 (4)	0.006 (4)	−0.003 (4)
C10	0.072 (6)	0.073 (5)	0.074 (6)	0.004 (5)	0.001 (5)	−0.018 (5)
C11	0.081 (7)	0.083 (6)	0.093 (7)	0.013 (5)	0.021 (5)	−0.011 (5)
C12	0.069 (6)	0.116 (8)	0.083 (7)	−0.011 (6)	0.019 (6)	0.007 (7)
C13	0.080 (7)	0.118 (8)	0.095 (8)	−0.043 (6)	0.002 (6)	0.011 (7)
C14	0.108 (8)	0.054 (5)	0.090 (7)	−0.010 (5)	0.015 (5)	−0.005 (4)
C15	0.079 (6)	0.069 (5)	0.071 (6)	0.014 (4)	0.003 (5)	−0.015 (5)
C16	0.067 (6)	0.061 (4)	0.057 (5)	0.010 (4)	−0.003 (4)	−0.001 (4)
C17	0.087 (7)	0.079 (6)	0.087 (7)	0.017 (5)	−0.018 (6)	−0.012 (5)
C18	0.092 (8)	0.120 (9)	0.104 (9)	0.044 (7)	−0.011 (6)	0.000 (7)
C19	0.098 (8)	0.142 (10)	0.081 (8)	0.002 (8)	−0.028 (6)	0.014 (8)
C20	0.112 (10)	0.121 (9)	0.104 (9)	−0.044 (8)	−0.018 (8)	0.001 (8)
C21	0.112 (9)	0.073 (6)	0.091 (7)	−0.010 (5)	−0.008 (6)	−0.011 (5)
S2	0.0663 (13)	0.0470 (10)	0.0872 (16)	0.0027 (9)	−0.0023 (11)	0.0021 (13)
O3	0.099 (5)	0.075 (4)	0.123 (5)	0.003 (3)	−0.005 (4)	−0.051 (4)
O4	0.085 (4)	0.080 (4)	0.099 (5)	0.026 (3)	0.004 (4)	0.034 (4)
N2	0.060 (4)	0.062 (4)	0.047 (4)	−0.001 (3)	−0.005 (3)	0.002 (3)
C22	0.065 (5)	0.054 (4)	0.063 (6)	0.002 (4)	−0.005 (4)	0.002 (4)
C23	0.088 (7)	0.062 (5)	0.089 (7)	−0.006 (5)	0.011 (5)	0.024 (5)
C24	0.083 (7)	0.090 (6)	0.084 (7)	−0.009 (5)	0.011 (5)	0.015 (6)
C25	0.064 (6)	0.093 (6)	0.058 (5)	0.004 (5)	−0.007 (5)	−0.007 (5)
C26	0.079 (7)	0.075 (5)	0.116 (8)	0.017 (5)	−0.008 (6)	0.020 (6)
C27	0.080 (7)	0.065 (5)	0.105 (7)	0.010 (5)	0.004 (6)	0.023 (5)
C28	0.070 (7)	0.165 (11)	0.117 (9)	0.030 (6)	0.000 (6)	0.013 (8)
C29	0.068 (6)	0.074 (5)	0.077 (6)	0.012 (4)	−0.008 (5)	0.007 (5)
C30	0.074 (6)	0.056 (4)	0.049 (5)	0.008 (4)	−0.004 (4)	0.001 (4)
C31	0.094 (8)	0.066 (5)	0.081 (6)	−0.001 (5)	−0.002 (5)	0.008 (5)
C32	0.110 (10)	0.112 (8)	0.091 (8)	−0.029 (7)	0.026 (7)	−0.003 (6)
C33	0.077 (7)	0.148 (9)	0.080 (7)	−0.007 (7)	0.014 (6)	−0.014 (7)
C34	0.094 (8)	0.120 (8)	0.095 (8)	0.019 (7)	0.021 (6)	0.008 (7)
C35	0.086 (7)	0.075 (6)	0.078 (6)	0.021 (5)	0.008 (5)	0.015 (4)
C36	0.082 (6)	0.057 (4)	0.071 (6)	0.006 (4)	−0.001 (5)	−0.014 (4)
C37	0.077 (6)	0.060 (5)	0.050 (5)	−0.007 (4)	−0.003 (4)	−0.017 (4)
C38	0.111 (8)	0.053 (5)	0.085 (7)	−0.012 (5)	−0.022 (6)	0.007 (4)
C39	0.084 (8)	0.107 (8)	0.095 (7)	−0.034 (6)	0.005 (6)	−0.011 (6)
C40	0.063 (6)	0.123 (8)	0.109 (8)	0.000 (7)	−0.005 (6)	−0.029 (7)
C41	0.084 (7)	0.086 (6)	0.094 (7)	0.008 (6)	−0.012 (5)	−0.010 (6)
C42	0.084 (6)	0.055 (5)	0.075 (6)	−0.003 (5)	−0.009 (5)	−0.004 (4)

### Geometric parameters (Å, °)

**Table:**

S1—O2	1.437 (6)	S2—O3	1.415 (6)
S1—O1	1.438 (6)	S2—O4	1.424 (6)
S1—N1	1.620 (6)	S2—N2	1.597 (6)
S1—C1	1.761 (7)	S2—C22	1.770 (7)
N1—C15	1.463 (9)	N2—C29	1.439 (10)
N1—C8	1.477 (9)	N2—C36	1.488 (9)
C1—C2	1.356 (9)	C22—C27	1.370 (10)
C1—C6	1.371 (10)	C22—C23	1.375 (10)
C2—C3	1.392 (10)	C23—C24	1.376 (11)
C2—H2	0.9300	C23—H23	0.9300
C3—C4	1.326 (11)	C24—C25	1.377 (12)
C3—H3	0.9300	C24—H24	0.9300
C4—C5	1.395 (11)	C25—C26	1.373 (12)
C4—C7	1.539 (11)	C25—C28	1.491 (12)
C5—C6	1.339 (11)	C26—C27	1.345 (12)
C5—H5	0.9300	C26—H26	0.9300
C6—H6	0.9300	C27—H27	0.9300
C7—H7A	0.9600	C28—H28A	0.9600
C7—H7B	0.9600	C28—H28B	0.9600
C7—H7C	0.9600	C28—H28C	0.9600
C8—C9	1.509 (11)	C29—C30	1.507 (11)
C8—H8A	0.9700	C29—H29A	0.9700
C8—H8B	0.9700	C29—H29B	0.9700
C9—C10	1.369 (10)	C30—C35	1.379 (10)
C9—C14	1.385 (10)	C30—C31	1.382 (11)
C10—C11	1.369 (11)	C31—C32	1.379 (13)
C10—H10	0.9300	C31—H31	0.9300
C11—C12	1.339 (13)	C32—C33	1.378 (14)
C11—H11	0.9300	C32—H32	0.9300
C12—C13	1.312 (14)	C33—C34	1.371 (14)
C12—H12	0.9300	C33—H33	0.9300
C13—C14	1.397 (13)	C34—C35	1.360 (12)
C13—H13	0.9300	C34—H34	0.9300
C14—H14	0.9300	C35—H35	0.9300
C15—C16	1.501 (11)	C36—C37	1.480 (11)
C15—H15A	0.9700	C36—H36A	0.9700
C15—H15B	0.9700	C36—H36B	0.9700
C16—C17	1.368 (11)	C37—C42	1.373 (10)
C16—C21	1.370 (11)	C37—C38	1.400 (11)
C17—C18	1.379 (13)	C38—C39	1.359 (12)
C17—H17	0.9300	C38—H38	0.9300
C18—C19	1.313 (14)	C39—C40	1.366 (14)
C18—H18	0.9300	C39—H39	0.9300
C19—C20	1.306 (15)	C40—C41	1.363 (13)
C19—H19	0.9300	C40—H40	0.9300
C20—C21	1.410 (14)	C41—C42	1.379 (11)
C20—H20	0.9300	C41—H41	0.9300
C21—H21	0.9300	C42—H42	0.9300
			
O2—S1—O1	118.5 (3)	O3—S2—O4	118.3 (4)
O2—S1—N1	107.3 (3)	O3—S2—N2	108.0 (4)
O1—S1—N1	108.3 (3)	O4—S2—N2	107.3 (3)
O2—S1—C1	108.9 (3)	O3—S2—C22	108.2 (4)
O1—S1—C1	106.0 (4)	O4—S2—C22	106.9 (4)
N1—S1—C1	107.4 (3)	N2—S2—C22	107.9 (3)
C15—N1—C8	117.7 (6)	C29—N2—C36	116.6 (6)
C15—N1—S1	121.2 (5)	C29—N2—S2	121.4 (5)
C8—N1—S1	117.1 (5)	C36—N2—S2	118.5 (5)
C2—C1—C6	118.8 (7)	C27—C22—C23	118.7 (7)
C2—C1—S1	122.4 (6)	C27—C22—S2	122.0 (6)
C6—C1—S1	118.7 (6)	C23—C22—S2	119.2 (6)
C1—C2—C3	119.2 (7)	C22—C23—C24	119.0 (8)
C1—C2—H2	120.4	C22—C23—H23	120.5
C3—C2—H2	120.4	C24—C23—H23	120.5
C4—C3—C2	121.6 (7)	C23—C24—C25	122.4 (8)
C4—C3—H3	119.2	C23—C24—H24	118.8
C2—C3—H3	119.2	C25—C24—H24	118.8
C3—C4—C5	118.8 (7)	C26—C25—C24	116.7 (8)
C3—C4—C7	121.9 (8)	C26—C25—C28	121.0 (9)
C5—C4—C7	119.1 (8)	C24—C25—C28	122.2 (9)
C6—C5—C4	119.6 (7)	C27—C26—C25	121.7 (8)
C6—C5—H5	120.2	C27—C26—H26	119.1
C4—C5—H5	120.2	C25—C26—H26	119.1
C5—C6—C1	121.8 (7)	C26—C27—C22	121.3 (8)
C5—C6—H6	119.1	C26—C27—H27	119.3
C1—C6—H6	119.1	C22—C27—H27	119.3
C4—C7—H7A	109.5	C25—C28—H28A	109.5
C4—C7—H7B	109.5	C25—C28—H28B	109.5
H7A—C7—H7B	109.5	H28A—C28—H28B	109.5
C4—C7—H7C	109.5	C25—C28—H28C	109.5
H7A—C7—H7C	109.5	H28A—C28—H28C	109.5
H7B—C7—H7C	109.5	H28B—C28—H28C	109.5
N1—C8—C9	112.8 (6)	N2—C29—C30	114.5 (6)
N1—C8—H8A	109.0	N2—C29—H29A	108.6
C9—C8—H8A	109.0	C30—C29—H29A	108.6
N1—C8—H8B	109.0	N2—C29—H29B	108.6
C9—C8—H8B	109.0	C30—C29—H29B	108.6
H8A—C8—H8B	107.8	H29A—C29—H29B	107.6
C10—C9—C14	117.8 (7)	C35—C30—C31	117.2 (8)
C10—C9—C8	120.6 (7)	C35—C30—C29	121.1 (8)
C14—C9—C8	121.5 (7)	C31—C30—C29	121.6 (7)
C11—C10—C9	120.3 (8)	C32—C31—C30	121.4 (9)
C11—C10—H10	119.8	C32—C31—H31	119.3
C9—C10—H10	119.8	C30—C31—H31	119.3
C12—C11—C10	120.3 (9)	C33—C32—C31	119.6 (10)
C12—C11—H11	119.8	C33—C32—H32	120.2
C10—C11—H11	119.8	C31—C32—H32	120.2
C13—C12—C11	122.0 (9)	C34—C33—C32	119.5 (10)
C13—C12—H12	119.0	C34—C33—H33	120.2
C11—C12—H12	119.0	C32—C33—H33	120.2
C12—C13—C14	119.3 (9)	C35—C34—C33	120.1 (10)
C12—C13—H13	120.4	C35—C34—H34	119.9
C14—C13—H13	120.4	C33—C34—H34	119.9
C9—C14—C13	120.2 (8)	C34—C35—C30	122.1 (9)
C9—C14—H14	119.9	C34—C35—H35	118.9
C13—C14—H14	119.9	C30—C35—H35	118.9
N1—C15—C16	113.1 (6)	C37—C36—N2	112.2 (6)
N1—C15—H15A	109.0	C37—C36—H36A	109.2
C16—C15—H15A	109.0	N2—C36—H36A	109.2
N1—C15—H15B	109.0	C37—C36—H36B	109.2
C16—C15—H15B	109.0	N2—C36—H36B	109.2
H15A—C15—H15B	107.8	H36A—C36—H36B	107.9
C17—C16—C21	118.1 (8)	C42—C37—C38	115.9 (8)
C17—C16—C15	122.8 (8)	C42—C37—C36	122.2 (8)
C21—C16—C15	119.0 (8)	C38—C37—C36	121.9 (8)
C16—C17—C18	120.4 (9)	C39—C38—C37	121.0 (8)
C16—C17—H17	119.8	C39—C38—H38	119.5
C18—C17—H17	119.8	C37—C38—H38	119.5
C19—C18—C17	120.5 (10)	C38—C39—C40	122.2 (9)
C19—C18—H18	119.7	C38—C39—H39	118.9
C17—C18—H18	119.7	C40—C39—H39	118.9
C20—C19—C18	121.3 (11)	C41—C40—C39	117.7 (9)
C20—C19—H19	119.3	C41—C40—H40	121.1
C18—C19—H19	119.3	C39—C40—H40	121.1
C19—C20—C21	120.7 (11)	C40—C41—C42	120.6 (9)
C19—C20—H20	119.6	C40—C41—H41	119.7
C21—C20—H20	119.6	C42—C41—H41	119.7
C16—C21—C20	118.8 (9)	C37—C42—C41	122.4 (8)
C16—C21—H21	120.6	C37—C42—H42	118.8
C20—C21—H21	120.6	C41—C42—H42	118.8
			
O2—S1—N1—C15	−29.7 (6)	O3—S2—N2—C29	26.3 (6)
O1—S1—N1—C15	−158.6 (5)	O4—S2—N2—C29	154.8 (6)
C1—S1—N1—C15	87.3 (6)	C22—S2—N2—C29	−90.4 (6)
O2—S1—N1—C8	173.4 (5)	O3—S2—N2—C36	−175.9 (5)
O1—S1—N1—C8	44.5 (6)	O4—S2—N2—C36	−47.4 (6)
C1—S1—N1—C8	−69.6 (6)	C22—S2—N2—C36	67.4 (6)
O2—S1—C1—C2	88.0 (7)	O3—S2—C22—C27	−85.8 (8)
O1—S1—C1—C2	−143.5 (7)	O4—S2—C22—C27	145.8 (7)
N1—S1—C1—C2	−27.9 (7)	N2—S2—C22—C27	30.7 (8)
O2—S1—C1—C6	−89.0 (7)	O3—S2—C22—C23	95.7 (7)
O1—S1—C1—C6	39.5 (7)	O4—S2—C22—C23	−32.7 (7)
N1—S1—C1—C6	155.1 (6)	N2—S2—C22—C23	−147.7 (6)
C6—C1—C2—C3	−1.2 (12)	C27—C22—C23—C24	3.4 (12)
S1—C1—C2—C3	−178.2 (6)	S2—C22—C23—C24	−178.1 (7)
C1—C2—C3—C4	4.9 (13)	C22—C23—C24—C25	−0.7 (14)
C2—C3—C4—C5	−5.3 (13)	C23—C24—C25—C26	−1.9 (14)
C2—C3—C4—C7	−179.8 (8)	C23—C24—C25—C28	−179.9 (9)
C3—C4—C5—C6	2.1 (14)	C24—C25—C26—C27	1.7 (14)
C7—C4—C5—C6	176.8 (8)	C28—C25—C26—C27	179.8 (10)
C4—C5—C6—C1	1.5 (14)	C25—C26—C27—C22	1.0 (15)
C2—C1—C6—C5	−1.9 (13)	C23—C22—C27—C26	−3.6 (13)
S1—C1—C6—C5	175.2 (7)	S2—C22—C27—C26	177.9 (8)
C15—N1—C8—C9	79.6 (8)	C36—N2—C29—C30	83.2 (8)
S1—N1—C8—C9	−122.7 (6)	S2—N2—C29—C30	−118.6 (6)
N1—C8—C9—C10	73.6 (10)	N2—C29—C30—C35	39.1 (11)
N1—C8—C9—C14	−109.7 (8)	N2—C29—C30—C31	−141.3 (7)
C14—C9—C10—C11	1.9 (13)	C35—C30—C31—C32	0.0 (13)
C8—C9—C10—C11	178.7 (8)	C29—C30—C31—C32	−179.7 (9)
C9—C10—C11—C12	−1.8 (14)	C30—C31—C32—C33	−1.7 (15)
C10—C11—C12—C13	2.0 (16)	C31—C32—C33—C34	2.8 (16)
C11—C12—C13—C14	−2.2 (16)	C32—C33—C34—C35	−2.1 (16)
C10—C9—C14—C13	−2.2 (12)	C33—C34—C35—C30	0.3 (16)
C8—C9—C14—C13	−178.9 (8)	C31—C30—C35—C34	0.7 (13)
C12—C13—C14—C9	2.3 (15)	C29—C30—C35—C34	−179.6 (9)
C8—N1—C15—C16	−83.4 (8)	C29—N2—C36—C37	−79.4 (8)
S1—N1—C15—C16	119.8 (6)	S2—N2—C36—C37	121.7 (7)
N1—C15—C16—C17	−41.2 (11)	N2—C36—C37—C42	−69.6 (11)
N1—C15—C16—C21	142.3 (8)	N2—C36—C37—C38	110.8 (8)
C21—C16—C17—C18	−2.5 (14)	C42—C37—C38—C39	3.5 (13)
C15—C16—C17—C18	−179.1 (9)	C36—C37—C38—C39	−176.9 (8)
C16—C17—C18—C19	2.9 (17)	C37—C38—C39—C40	−2.0 (15)
C17—C18—C19—C20	−3.0 (18)	C38—C39—C40—C41	−0.8 (15)
C18—C19—C20—C21	2.7 (18)	C39—C40—C41—C42	2.0 (14)
C17—C16—C21—C20	2.2 (14)	C38—C37—C42—C41	−2.4 (12)
C15—C16—C21—C20	178.9 (8)	C36—C37—C42—C41	178.0 (8)
C19—C20—C21—C16	−2.3 (16)	C40—C41—C42—C37	−0.3 (14)

### Hydrogen-bond geometry (Å, °)

**Table:**

*D*—H···*A*	*D*—H	H···*A*	*D*···*A*	*D*—H···*A*
C8—H8a···O1^i^	0.97	2.58	3.456 (9)	151
C36—H36a···O4^i^	0.97	2.51	3.404 (9)	154

Symmetry codes: (i) *x*, *y*+1, *z*.
